# Testing Vulcanizers

**Published:** 1865-01

**Authors:** W. P. Haywood


Testing Vulcanizers.—*By W. P. Haywood.*—Fill the boiler as full of water as it will hold, screw on the lid, and, if there is a valve, fasten down or load as heavy as it will ever require to be at the highest point of the mercury for vulcanizing; then apply heat, which will cause the water to expand, long before it reaches its boiling point, with great force, and if there is a leak in the packing, or the boiler otherwise defective, the water will ooze out.

